# Supplementary material for: Integrative Neoepitope Discovery in Glioblastoma via HLA Class I Profiling and AlphaFold2-Multimer
Source: Biomedicines. 2025 Nov 5;13(11):2715. doi: 10.3390/biomedicines13112715 (PMC12650544; doi:10.3390/biomedicines13112715)
Supplement: Supplementary file 1 [file biomedicines-13-02715-s001.zip › biomedicines-3916327-supplementary.pdf]

MDPI

biomedicines

Supplementary material

Integrative Neoepitope Discovery in Glioblastoma via HLA Class I Profiling and AlphaFold2-Multimer

Raquel Francés [1], Jenny Bonifacio-Mundaca [2], Íñigo Casafont [1], Christophe Desterke [3,*] and Jorge Mata-Garrido [1,*]

[1] Anatomy & Cell Biology Department, School of Medicine, University of Cantabria and Nanomedicine Group, Valdecilla-IDIVAL Institute, 39011 Santander, Spain
[2] National Tumor Bank, Department of Pathology, National Institute of Neoplastic Diseases, Surquillo 15038, Peru
[3] Faculté de Médecine du Kremlin Bicêtre, Université Paris-Sud, Université Paris-Saclay, 94270 Le Kremlin-Bicêtre, France
* Correspondence: christophe.desterke@inserm.fr; jmatag@unican.es

Supplementary Table S1. Public databases and bioinformatic resources used in this study. This table summarizes all publicly available databases and repositories utilized for variant annotation, allele reference sequences, and integrative analysis of glioblastoma genomic and immunogenomic data. For each resource, the table provides the version employed, a brief description of its content or analytical purpose, and the corresponding web address. Databases include repositories of somatic mutations (COSMIC, TCGA), germline variant filtering (dbSNP), transcript annotation (MANE), HLA allele reference sequences (IPD-IMGT/HLA), and genomic visualization or coordinate mapping (UCSC Genome Browser). All resources were accessed between January and March 2025, and the latest stable releases available during that period were used.

Database	Description	URL	Reference
dbSNP v155	Repository of common human genetic polymorphisms used to exclude germline variants.	https://www.ncbi.nlm.nih.gov/snp	[28]
COSMIC v102	Catalogue of somatic mutations in human cancers.	https://cancer.sanger.ac.uk/cosmic	[30]
MANE v1.2	Matched NCBI/Ensembl transcript set for consistent annotation.	https://www.ensembl.org/info/genome/genebuild/mane.html	[29]
UCSC Genome Browser	Data portal for genomic coordinates and annotation.	https://genome.ucsc.edu/	[27]
TCGA	Pan-cancer genomic and clinical dataset used for somatic mutation calls.	https://portal.gdc.cancer.gov/	[26]
IPD-IMGT/HLA	Reference repository for HLA allele sequences and nomenclature.	https://www.ebi.ac.uk/ipd/imgt/hla/	[33]

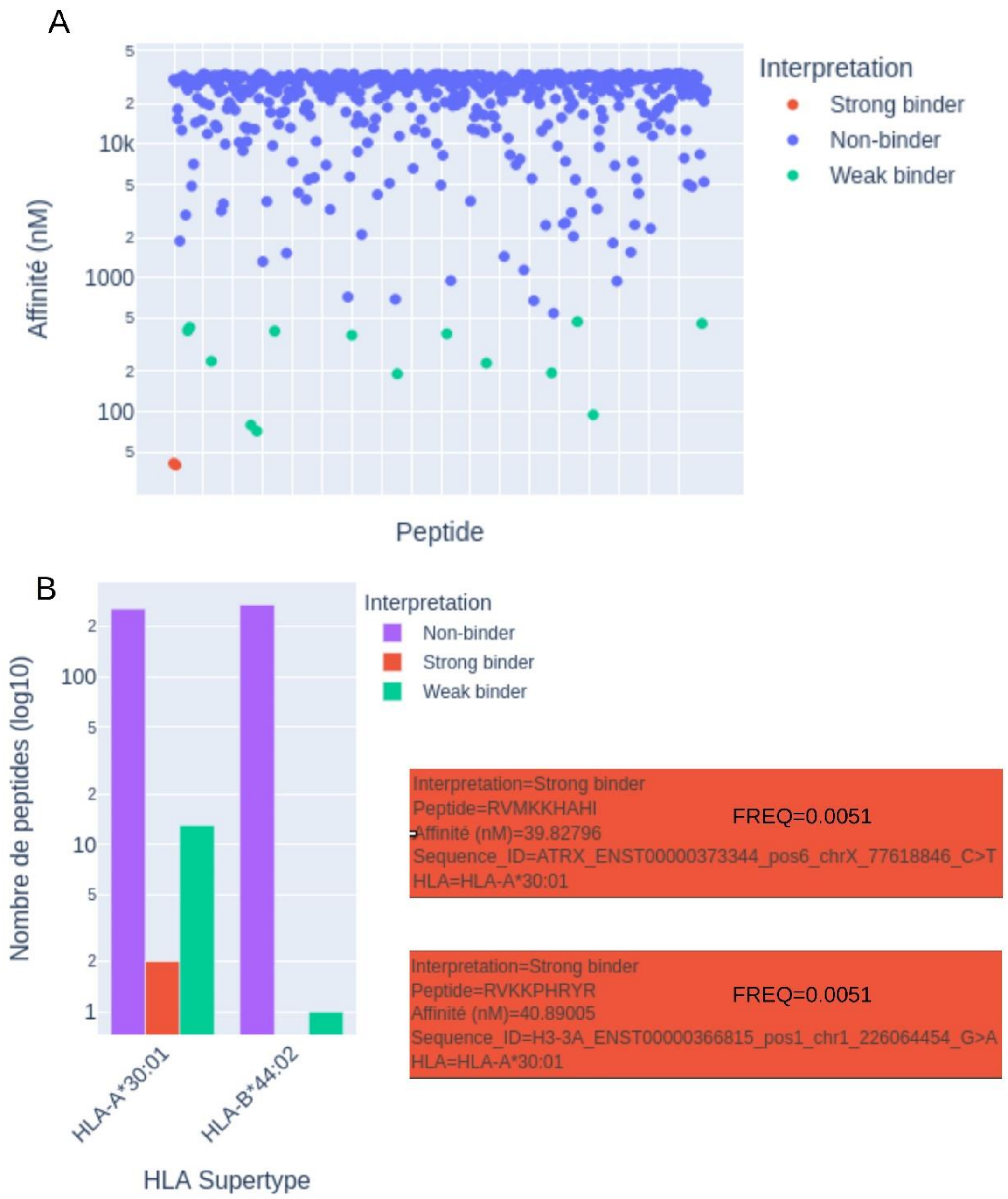

Supplementary Figure S1. GBM Peptide Binding Affinities for HLA HLA-A30:01 and HLA-B44:02 alleles. (A) Scatter plot showing the predicted binding affinities (nM) of 9-mer peptides for HLA HLA-A30:01 and HLA-B44:02 alleles. Each point represents a peptide, color-coded by binding strength: red for strong binders, green for weak binders, and blue for non-binders. (B) Bar chart displaying the log10-scaled count of peptides associated with HLA-A30:01 and HLA-B44:02 supertypes. Bars are color-coded by binding interpretation. Red boxes highlight two strong-binding peptides with low affinity values and frequency, linked to distinct sequence variants and the HLA-A*30:01 allele.
